# Quantitative EEG in Cognitive Neuroscience

**DOI:** 10.3390/brainsci11040517

**Published:** 2021-04-19

**Authors:** Yvonne Höller

**Affiliations:** Faculty of Psychology, University of Akureyri, 600 Akureyri, Iceland; yvonne@unak.is

Quantitative electroencephalography (EEG) distinguishes itself from clinical EEG by the application of mathematical approaches and computer scientific methods. Its quantitative nature is largely owned to digital signal analysis, enabling a quantitative description of the waveforms which are obtained when we record an EEG. Indeed, digital signal processing has opened a new world for cognitive neuroscientists. The EEG allows to measure precisely in time when cognitive processes begin and end. Analyzing dynamics and signal similarities quantitatively reveal insights into propagation patterns of information and most likely also the exchange of neural messages. Cognitive research in psychology—but also in neurological fields of application—take advantage of classical and advanced methods in quantitative EEG research.

After much hype about connectivity, brain computer interfaces that promise mind-reading based on artificial intelligence and easy-to use mobile EEG systems for entertainment and gaming, critical views on replicability of EEG research in cognitive neuroscience are highly warranted to restore awareness of common methodological pitfalls and to rise the reputation of the field.

One of the most classical methods of quantitative EEG in cognitive neuroscience are event related potentials (ERPs). They are obtained by repeating a certain stimulus and averaging the brain response that is time-locked to this stimulus’ onset. Brain computer interfaces are often based on the P300, a component of the ERP that reliably responds to attentional effects. Although this and similar components are supposed to be used for brain computer interfaces that aim at making life easier for people with disabilities, possibly due to brain damage, the effect of pathology on the validity of these markers has rarely been assessed. In this Special Issue, Lytaev and Vatamaniuk demonstrate variations in latency and amplitude related to brain pathology [1]; thus, emphasizing the need to probe brain computer interfaces in target populations of patients with pathophysiological conditions. This is important as findings from healthy populations might not be easily transferrable to those populations. Patient populations present with abnormalities in brain activity being related to the primary injury, but also to neuroplasticity that emerges at the chronic stage of the condition.

The EEG as a neuroscientific method has several important advantages. One of them is being portable. Field studies would not be possible with magnetic resonance imaging or magnetoencephalography. However, mobile EEG devices have been viewed critically because of their low signal-to-noise ratio and, thus, their poor reliability. Especially recordings in the field might be biased by the uncontrolled environment. In this Special Issue, Edwards and Truillo probe the validity limits of the EEG by directly contrasting a mobile EEG being recorded under controlled laboratory conditions with recordings in an uncontrolled outdoor environment [2]. Assessment of EEG spectral power responses to cognitive stimulation (number sense, attention, memory, executive function) showed that significant effects could be recorded under both recording conditions. Lower frequency bands up to alpha (8–13 Hz) were not affected by the environment, but beta-band power was potentially affected at resting state. This finding is encouraging as also neurofeedback technology may be employed under varying conditions. For example, Pérez-Elvira et al., used individual alpha peak frequency as a biomarker for neurofeedback training in adolescents with learning disabilities [3]. In their study, this biomarker was used to identify patients who might be more likely to respond to the neurofeedback training. In order to be used in clinical practice to this end, a biomarker must be reliable and replicable. The EEG is still mostly used in a clinical field of application, where qualitative assessment is today’s standard. Nevertheless, even in the clinical environment quantitative methods are introduced more and more, as the hope is that they will render clinical applications more reliable and that they will ultimately make clinical EEG assessment more time-efficient by introducing automation.

Another advantage of the EEG as a neuroscientific method is that it is not harmful and can, therefore, be used in vulnerable groups, such as in the study of cognitive processes very early in a human’s life. Lang et al. conducted two studies in newborns at the age of 2–5 weeks after birth [4,5]. With this study setup, it was possible to show that the brain activity in newborns shows a distinctive reaction to the maternal voice compared to an unfamiliar voice, but that a prenatally repeatedly presented rhyme did not evoke such a familiarity effect. However, the prenatal daily stimulation with a maternal spoken nursery rhyme was found to evoke a certain type of memory, such that a re-exposure to this nursery rhyme after birth had a calming effect; polysomnography and video-monitored high-density EEG revealed fewer waking states, more time spent in deep sleep and lower heartrates as response to the familiar nursery rhyme. Both studies represent a hint to very early memory formation, possibly even pre-natal memory.

The EEG can also be understood in a wider sense, when we move from the classical scalp-EEG to invasive methods. The medical necessity to implant electrodes in order to measure the EEG invasively is a very unique opportunity for neuroscientists to study the activity of specific brain regions deep below the neocortex. Additionally, addressing medical questions with methods of quantitative EEG and paradigms from cognitive neuroscience is also yielding important insights that might be of relevance for future medical decision making. Basic questions about the validity of conclusions on effective neural signaling between brain regions can also be addressed in such a scenario. Based on a sample of pre-surgical patients with implanted electrodes, Thomschewski et al. critically asked whether neural activity in high frequency bands at 80–249 Hz is coherent between distant brain regions [6]. Their study revealed that coherent activity of high frequency oscillations between the prefrontal cortex and the hippocampus was not significantly altered during completion of a virtual spatial navigation task. It is possible that this band of high frequency activity is reflecting rather local circuit activities rather than long-distance communication. Locally refined activity can also be examined by quantitative EEG methods down to the single unit level. The activity of single neurons can be examined when microelectrodes are implanted in patients with epilepsy, undergoing pre-surgical evaluation. Derner et al. analyzed firing rates of single neurons in response to binaural versus monaural 5 Hz stimulation [7]. A negative correlation between beat stimulation-related firing rate differences and memory-related firing rates suggested that beat stimulation may be shifting baseline firing levels. Indeed, the authors reported that this shift in firing rate was associated with increased memory performance for binaural vs. monaural beats.

Quantitative EEG combined with cognitive tasks is established in cognitive neuroscience and has been introduced to clinical decision making, a domain that requires reliable and replicable signals. These are indeed the challenges of quantitative EEG in cognitive neuroscience, as the replication crisis has reached this field like many others. Analysis methods in quantitative EEG are often described as a “jungle”, where new methods are established rapidly and sometimes unfortunately adapted by unexperienced users. Poor replicability can easily arise from the wide variety of methods that can be combined and the pitfalls that can be met without the necessary understanding of statistics and digital signal processing. Furthermore, sample sizes in EEG research are traditionally wrong. Some decades ago, the rule of thumb was that an EEG study should include around 20 participants. However, this number bears any statistical argument, as the power of such a study varies with the effect size of the particular biomarker and comparison. Some researchers have adapted more lenient views and report results on considerably smaller samples, which require the use of robust, non-parametric statistics. Other researchers have acknowledged the problem and aim for larger samples in their studies, at the order of 100 participants or more. Especially in machine learning, this is still a small number.

For boosting the field of quantitative EEG in cognitive neuroscience, it will be necessary to plan adequately powered studies with large sample sizes. Moreover, longitudinal research with repeated EEG recordings in order to proof the repeatability of findings with specific biomarkers and studies that employ robust statistical methods that were tested on EEG data can propel this field forward.
